# WPK5, a Novel Kunitz-Type Peptide from the Leech *Whitmania pigra* Inhibiting Factor XIa, and Its Loop-Replaced Mutant to Improve Potency

**DOI:** 10.3390/biomedicines9121745

**Published:** 2021-11-23

**Authors:** Yi-Zheng Zheng, Xiao-Ru Ji, Yun-Yang Liu, Shuai Jiang, Xiang-Ying Yu, Zhi-Ping Jia, Yue Zhao, Jun-Qiao Zhang, Jia-Li Zhang, Yi Kong

**Affiliations:** School of Life Science and Technology, China Pharmaceutical University, 24 Tong Jia Street, Nanjing 210009, China; 3219030707@stu.cpu.edu.cn (Y.-Z.Z.); 1822030732@stu.cpu.edu.cn (X.-R.J.); 1821030696@stu.cpu.edu.cn (Y.-Y.L.); 3219030711@stu.cpu.edu.cn (S.J.); 3220030462@stu.cpu.edu.cn (X.-Y.Y.); 3219030696@stu.cpu.edu.cn (Z.-P.J.); 2020191042@stu.cpu.edu.cn (Y.Z.); 2020202406@stu.cpu.edu.cn (J.-Q.Z.); 3320031529@stu.cpu.edu.cn (J.-L.Z.)

**Keywords:** transcriptome, *Whitmania pigra*, Kunitz-type peptides, Loop replacement, FXIa inhibitor, Antithrombosis

## Abstract

Kunitz-type proteins or peptides have been found in many blood-sucking animals, but the identity of them in leeches remained elusive. In the present study, five Kunitz-type peptides named WPK1-WPK5 were identified from the leech *Whitmania pigra*. Recombinant WPK1-WPK5 were expressed in *Pichia pastoris* GS115, and their inhibitory activity against Factor XIa (FXIa) was tested. WPK5 showed inhibitory activity against FXIa with an IC_50_ value of 978.20 nM. To improve its potency, the loop replacement strategy was used. The loop 1 (TGPCRSNLER) and loop 2 (QYGGC) in WPK5 were replaced by loop 1 (TGPCRAMISR) and loop 2 (FYGGC) in PN2KPI, respectively, and the resulting peptide named WPK5-Mut showed an IC_50_ value of 8.34 nM to FXIa, which is about 100-fold the potency of FXIa compared to that of WPK5. WPK5-Mut was further evaluated for its extensive bioactivity in vitro and in vivo. It dose-dependently prolonged APTT on both murine plasma and human plasma, and potently inhibited FeCl_3_-induced carotid artery thrombosis in mice at a dose of 1.5 mg/kg. Additionally, WPK5-Mut did not show significant bleeding risk at a dose of 6 mg/kg. Together, these results showed that WPK5-Mut is a promising candidate for the development of an antithrombotic drug.

## 1. Introduction

Kunitz-type serine protease inhibitors are ubiquitous and were found in many organisms, including animals, plants and microorganisms [1]. The classical Kunitz-type peptide is composed of 50–60 amino acids and adopts a conservative structural folding method which include two antiparallel β-sheets and one α-helix. The conformational stability of Kunitz-type peptide is maintained through three highly conserved disulfide bridges (Cys^1^-Cys^6^, Cys^2^-Cys^4^, Cys^3^-Cys^5^) [2,3,4], and the inhibitory specificity of the Kunitz-type peptide varies with the specific amino acid of the reaction site. In vertebrates, Kunitz inhibitors play a major role in inflammation, blood clotting and digestion, while in invertebrates, they participate in development of reproductive system, regulation of collagen synthesis and other physiology process [5,6]. Most Kunitz-type inhibitors in blood-sucking arthropods act as anticoagulant factors [7].

Coagulation factor XI (FXI) is a zymogen of FXIa [8], which can be activated by FXIIa. FXIa promotes fibrin stabilization and thrombin production through a sequential activation of FIX and FX [9,10]. Epidemiological studies have shown that patients with severe deficiency of FXI have a relatively low risk of ischemic stroke and deep vein thrombosis while without spontaneous bleeding [11,12]. Similarly, high levels of FXI could increase women’s risk of deep vein thrombosis [13], stroke [14,15,16], myocardial infarction [17], and cardiovascular disease [18]. In some animal models of arterial and venous thrombosis, FXI knockout mice showed strong antithrombotic activity without excessive bleeding [19,20,21,22], and the similar phenomena were observed in rats [23], rabbits [24] and baboons [25,26]. Therefore, it is possible to develop safe and effective anticoagulants with low risk of bleeding when targeting FXI/FXIa. The current FXI/FXIa inhibitors can be divided into seven categories, including peptides, peptidomimetic active site inhibitors, polyglycosaminoglycans (GAGs), non-sugar GAG mimics, antibodies, antisense oligonucleotides (ASO) and aptamers [27]. Among them, peptide inhibitors such as PN2KPI [28], Fasxiator [29] and Desmolaris [30], contain Kunitz domain which can bind to serine proteases in a substrate-like manner and inhibit their activities.

The leech is a kind of arthropod with important medicinal value in traditional and modern medicine. There are a large number of antithrombotic components in the salivary glands of leech, and they are divided into two categories. One is anticoagulant proteins, including thrombin inhibitors and FXa inhibitors; the other is anti-platelet aggregation proteins. The thrombin inhibitors include Hirudin [31], Haemadin [32] and Theromin [33], FXa inhibitors include Therostasin [34], Antistasin [35] and Lefaxin [36], and anti-platelet aggregation proteins include Calin, LAPP (Leech anti-platelet protein), Decorsin and Ornatin [37,38,39,40]. However, inhibitors targeting FXI from leeches have not been reported.

In this study, five Kunitz-type peptides were obtained from the salivary gland transcriptome of the leech *Whitmania pigra*. Recombinant WPK1-WPK5 were expressed in *Pichia pastoris* GS115 and their inhibitory activity against FXIa were tested. Among them, WPK5 showed strong inhibitory activity to FXIa while showing negligible inhibition to other proteases of the coagulation cascade. Then, loop replacement was used to improve the potency of WPK5 to FXIa. The selectivity of WPK5-Mut to other coagulation factors, in vitro anticoagulant and in vivo antithrombotic activities were further evaluated.

## 2. Materials and Methods

### 2.1. Materials and Reagents

Factor Xa, Thrombin, Trypsin, Factor XIIa, Pefachrome FXIIa/TH 5253 (H-D-CHA-Gly-Arg-pNA·2AcOH), CS-11(22) (Bz-Ile-Glu(γOCH3)-Gly-Arg-pNa and Bz-Ile-Glu(γOH)-Gly-Arg-pNa), CS-31(02) (D-Pro-Phe-Arg-pNa, 2HCl), CS-21(66) (p-Glu-Pro-Arg-pNa·HCl) and CS-01(38) (H-D-Phe-Pip-Arg-pNa, 2HCl) were from Hyphen-Biomed, (Neuville-Sur-Oise, France); HFXIa was from Enzyme Research Laboratories, (South Bend, IN, USA); Kallikrein was from AssayPro LLC, (St. Charles, MI, USA); APTT and PT reagent were from STEELLEX (Beijing, China); FeCl_3_ was from Sin-opharm Chemical Reagent Co, Ltd. (Shanghai, China); All other chemicals used in this study were of analytical grade.

### 2.2. Animals

C57BL/6J mice (male, 20–25 g) were purchased from Comparative Medicine Centre of Yangzhou University (Jiangsu province, China). All animals were housed under controlled temperature (23–25 °C), humidity (30–40%) and light (12 h light, 12 h dark), with free access to food and water. All animals were tested after one week of adaptive feeding. Human venous blood was obtained from healthy adult volunteers in accordance with the Declaration of Helsinki. All protocols used were approved by the Ethics Committee of the China Pharmaceutical University.

### 2.3. Screening of Kunitz-Type Peptides

The transcriptome of the salivary gland of the leech *Whitmania pigra* was built previously in our lab [41]. Kunitz-type peptides were searched in the annotated transcriptome library with a “Kunitz” Keywords:

### 2.4. Clone, Expression and Purification of Kunitz-Type Peptides

The gene sequences of WPK1-WPK5 and WPK5-Mut were synthesized and then enriched by PCR. pPIC9K was cleaved with EcoRI and NotI to obtain a linearized vector, and the target gene fragment was recombined into pPIC9K by homologous recombination to obtain a full-length plasmid. Then, the target gene in pPIC9K was confirmed by DNA sequencing. The plasmid contains an open reading frame, α factor signal peptide sequence, a 57 amino acid Kunitz-type peptide sequence and a C-terminal 6 *(His) tag. The plasmids were linearized with the SacI restriction enzyme (TaKaRa). The linearized plasmids were then transformed into *Pichia pastoris* GS115 cells using an Electroporator (Bio-Rad, Hercules, CA, USA). The positive clones were cultured in BMMY medium, and the expression was induced by adding 1% (*v*/*v*) methanol for 72 h. The fermentation broth was purified by column previously loaded with UniNTA-80Ni (Nanomicro, Suzhou, China), and target peptide was eluted by 20 mmol/L PBS including 200 mmol/L imidazole.

### 2.5. HPLC and LC-Q-TOF-MS Analysis

WPK5 or WPK5-Mut was loaded to RP-HPLC on a C_18_ column (4.6 mm × 250 mm, Agilent Technologies, Santa Clara, CA, USA). Eluent A consisted of 0.1% TFA in distilled water (*v*/*v*), and eluent B consisted of 0.1% TFA in 100% acetonitrile (*v*/*v*). Elution was carried out according to the following process: 0–20 min, B 10–70%; 20–22 min, B 70–90%; and 22–25 min, B 90% at a flow rate of 1 mL/min and the absorbance of the elution fractions was monitored at 214 nm. WPK5 and WPK5-Mut were qualitatively analyzed by using LC-Q-TOF-MS. The analysis was performed on an Agilent Zorbax SB-C_18_ (4.6 mm × 250 mm) and the mobile phase consisted of 0.1% formic acid in MilliQ water (solvent A) and 0.1% formic acid in 100% acetonitrile (solvent B) at a flow rate of 0.5 mL/min. The mobile-phase gradient (10–70% B) was applied. Source nitrogen gas temperature was 325 °C, sheath gas flow was 12 L/min, and nebulizer pressure was 40 psig. Voltages were set at 4000 (capillary) and 175 V (fragmentor). Positive ions were acquired in the range of 100–3200 *m*/*z* for MS scans. Internal mass correction was enabled by using two reference masses at 121.0509 and 922.0098 *m*/*z*. Data acquisition and instrument control were performed using Agilent MassHunter Workstation software (B.06.01 SPI).

### 2.6. FXIa Inhibitory Activity Test of Kunitz-Type Peptides

The inhibitory activity of purified Kunitz-type peptides to FXIa were tested as previously described [42]. In brief, FXIa (100 μL) diluted with buffer TBS-BSA (50 mM Tris, 0.75 M NaCl, 1.5% BSA, pH 7.4) was pre-incubated with 50 μL different concentrations of peptides for 60 min at 37 °C, followed by the addition of 50 μL of the chromogenic substrate CS-21(66). A microplate reader (FEL-1) was used to continuously monitor the hydrolysis of the substrate at 405 nm for 60 min. The slope of the absorbance-time curve was used to calculate the inhibitory activity of Kunitz-type peptide. The inhibitory rate was calculated according to the following equation:

Inhibitory rate (%) = (V_0_ − V_i_)/V_0_ × 100 (%)

where V_0_ represents the slope of the control and V_i_ represents the slope of different concentrations of Kunitz-type peptide. IC_50_ was defined as the concentration of inhibitor required to inhibit the activity of FXIa by 50%.

### 2.7. Protease Selectivity Assay

WPK5 or WPK5-Mut and serine protease (trypsin (0.5 nM), FXIIa (1 nM), kallikrein (0.5 nM), FXa (2.5 nM), or thrombin (1.42 nM)) were mixed with different molar ratios in TBS-BSA, and then incubated for 60 min at 37 °C. The chromogenic substrate (CS-11(22) (0.44 mM), TH 5253 (0.25 mM), CS-31(02) (0.25 mM), CS-11(22) (0.44 mM), CS-01(38) (0.10 mM)) was added into the corresponding protease well, and absorbance at 405 nm was then continuously tested for 1 h using a microplate reader (FEL-1).

### 2.8. APTT and PT Assay

For APTT assay, 40 μL PPP and 10 μL different concentrations of WPK5 or WPK5-Mut were added to the test cup and incubated at 37 °C for 3 min, followed by the addition of 50 μL APTT reagent, and then incubated for more 3 min. Finally, 50 μL of pre-warmed CaCl_2_ solution was added to initiate clotting and the fibrin formation time was recorded in duplicate using Semi-automatic coagulation analyzer (SC40 (LG-PABER-I), STEELLEX, Beijing, China). For PT assay, 40 μL PPP and 10 μL different concentrations of WPK5 or WPK5-Mut were added to the test cup and incubated at 37 °C for 3 min, followed by the addition of 100 μL PT reagent to initiate clotting and the fibrin formation time was recorded in duplicate using Semi-automatic coagulation analyzer (SC40 (LG-PABER-I), STEELLEX, Beijing, China).

### 2.9. Antithrombotic Activity Assay

The in vivo antithrombotic activity of WPK5-Mut was evaluated by using a FeCl_3_-induced carotid artery thrombosis model in mice [43]. Mice (C57BL/6J, males, 18–22 g) were anesthetized by intraperitoneal injection of chloral hydrate (5%, 10 mL/kg) and then placed in a supine position on a heating pad (37 °C) to maintain body temperature. An incision was made in the middle of the mouse’s neck and the fascia and muscle were gradually separated to expose the carotid artery. A rubber strip (4 × 10 mm) was placed under the artery to separate it from the surrounding tissues, and mice were injected heparin sodium (2.7 mg/kg), WPK5-Mut (0.75, 1.5 and 3 mg/kg) or saline through the tail vein and placed under a laser speckle contrast imaging (moor FLPI-2, Moor Instruments Limited, Millwey, Axminster, Devon, EX13 5HU, UK) to observe the carotid blood flow. Ten min later, a filter paper strips (1 mm × 2 mm) soaked with 6% FeCl_3_ solution was placed on the carotid artery for 3 min and then removed, the blood flow was observed for 30 min. The occlusion time was defined as the time from the removal of the FeCl_3_ saturated filter paper to the blockage of the vessel for at least 3 consecutive min [44]. If a vessel is blocked at a certain time for 3 consecutive min, this time is recorded as occlusion time, and if no blockage occurs within 30 min, the occlusion time is recorded as 30 min. The blood flow was analyzed by moorFLPI ReviewV50 software.

### 2.10. Tail Bleeding Time Assay

To assess the bleeding risk of WPK5-Mut, a tail cutting assay was used [45]. The mice were anesthetized by intraperitoneal injection of 5% chloral hydrate (10 mL/kg), and heparin sodium (2.7 mg/kg), WPK5-Mut (3 and 6 mg/kg) or saline were injected by tail vein, after fifteen min the tail of the mouse was cut about 3 mm from the tail tip and immersed in 37 °C saline. A timer was used to record the accumulated blood flow time after cutting the tail tip (the timing was suspended when the blood flow stopped, and continued when the bleeding appeared again, and if the bleeding time was >20 min then it was recorded as 20 min).

### 2.11. Statistical Analysis

Data were analyzed by using GraphPad Prism 6 (GraphPad Software, Inc., La Jolla, CA, USA). Results were expressed as mean ± SD values. The statistical differences among multiple-samples were evaluated by using one-way ANOVA analysis followed by Dunnett’ s multiple comparison test. *p* < 0.05 was considered to be statistically significant.

## 3. Results

### 3.1. Five Kunitz-Type Peptides Were Identified from the Salivary Gland of the Leech Whitmania Pigra

**Figure 1:**
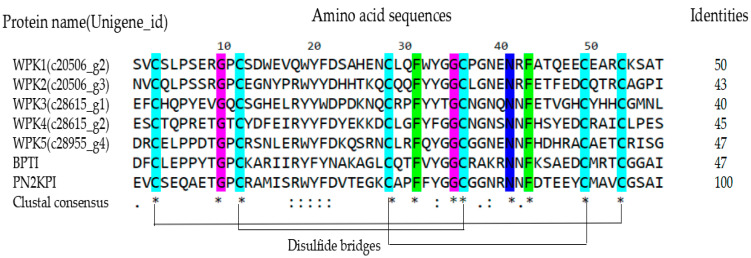
Clustal alignment of amino acid sequence of WPK1-WPK5, BPTI and PN2KPI. Conservative cysteine is marked with a light blue background. The disulfide bridges were shown by solid lines. “*” indicates positions with fully conserved residue. “:” indicates positions with similar properties and “.” indicates positions with weak similar properties.

The transcriptome of the leech *Whitmania pigra* salivary gland has been built in our lab [41]. Five Kunitz-type peptides named as WPK1-WPK5 were recognized from the annotated transcriptome data, which contained 57 amino acids and three disulfide bridges. All this peptides have more than 40% identities aligned with PN2KPI (Figure 1).

### 3.2. Clone, Expression, and Purification of WPK1-WPK5 and WPK5-Mut

WPK1-WPK5 and WPK5-Mut were expressed in *Pichia pastoris* GS115, and purification by UniNTA-80Ni column. Tricine-SDS-PAGE analysis showed that there is one band. The purity of WPK5 or WPK5-Mut was more than 95% by HPLC analysis (Figure A2A,B). LC-Q-TOF-MS showed that the molecular weight (MW) of WPK5 was 7400.2580 ± 0.0017 Da (Figure A2C), which is identical to the theoretical MW of 7400.25 Da. The determined MW of WPK5-Mut was 7378.2597 ± 0.0071 Da (Figure A2D), which is identical to the theoretical MW of 7378.25 Da.

### 3.3. WPK5 Is a FXIa Inhibitor

**Figure 2:**
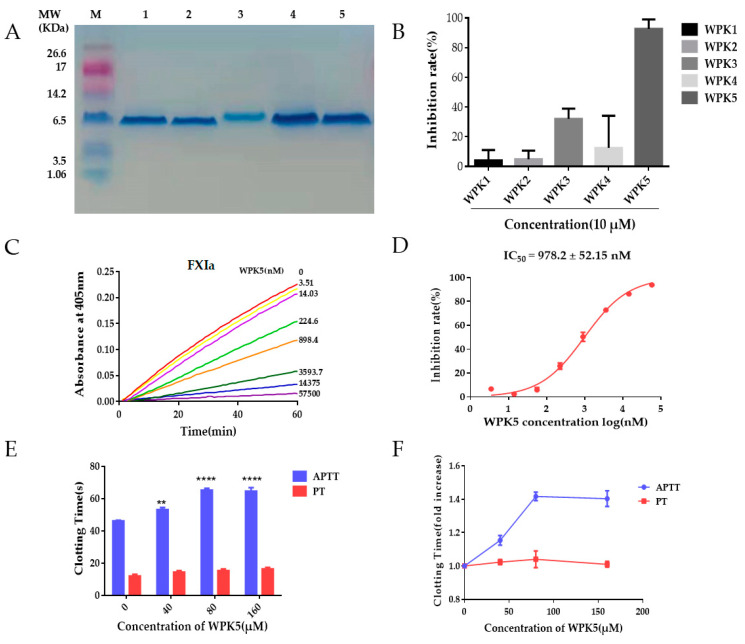
WPK5 is a FXIa inhibitor. (**A**) Tricine-SDS-PAGE of WPK1-WPK5, Lane M: Marker, Lane 1-5: WPK1-WPK5. (**B**) Inhibitory activity of WPK1-WPK5 to FXIa. (**C**) Inhibitory activity of WPK5 to FXIa. Reaction started by addition of CS-21 (66) (250 μM) to a mixture containing WPK5 (0–57500 nM) and FXIa (0.5 nM). Substrate was hydrolysed and monitored at 405 nm for 1 h. (**D**) Determination of IC_50_ of WPK5. Plot of inhibition rate (%) vs. log (WPK5 concentrations) was fitted by nonlinear regression. (**E**,**F**) Effect of WPK5 on APTT and PT of murine plasma. Data presented as mean ± SD of three independent experiments. **** *p* < 0.0001 versus vehicle, ** *p* < 0.01 versus vehicle, analyzed by one-way ANOVA followed by Dunnett’s multiple comparison test.

**Figure A1:**
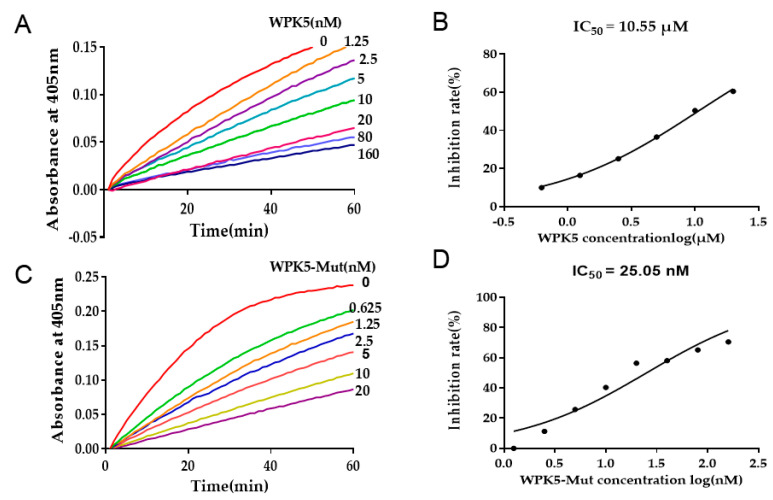
WPK5 and WPK5-Mut inhibited against trypsin. (**A**) Inhibitory activity of WPK5 to trypsin. WPK5 (0–160 μM) incubated for 60 min with trypsin (0.5 nM) followed by addition of CS-11(22) (0.44 mM) to start reaction. Substrate was hydrolyzed, and monitored at 405 nm for 60 min. (**B**) Determination of IC_50_ of WPK5. (**C**) Inhibitory activity of WPK5-Mut to trypsin. WPK5-Mut (0–20 nM) incubated for 60 min with trypsin (0.5 nM) followed by addition of CS-11(22) (0.44 mM) to start reaction. Substrate was hydrolyzed and monitored at 405 nm for 60 min. (**D**) Determination of IC_50_ of WPK5-Mut. Plot of inhibition rate (%) vs. log WPK5/WPK5-Mut concentrations fitted by nonlinear regression.

**Figure A2:**
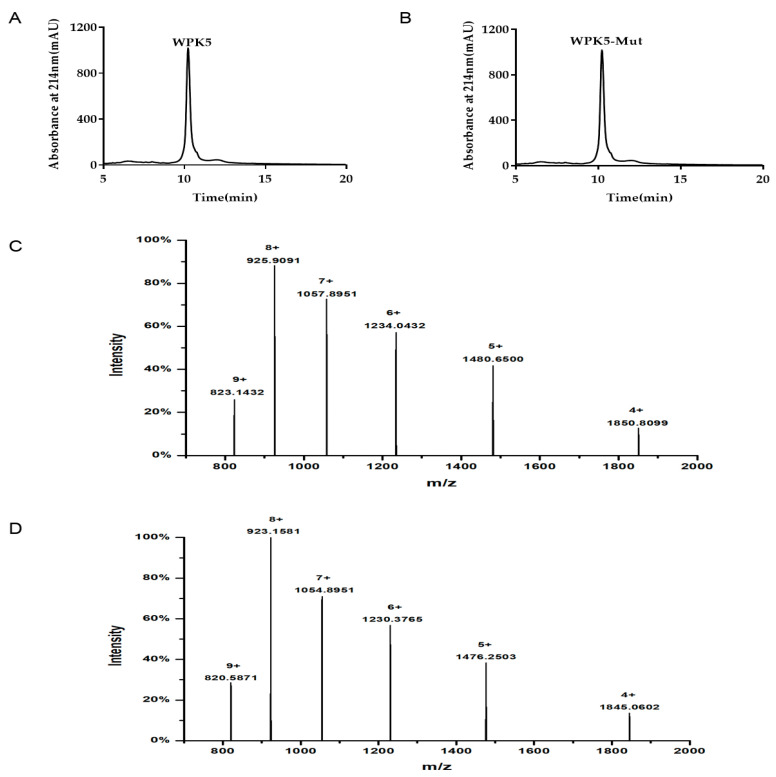
Characterization of WPK5 and WPK5-Mut. (**A**) HPLC analysis of WPK5. (**B**) HPLC analysis of WPK5-Mut. (**C**) LC-Q-TOF-MS analysis of WPK5. (**D**) LC-Q-TOF-MS analysis of WPK5-Mut.

Inhibitory activity of WPK1-WPK5 against FXIa was tested. At the concentration of 10 μM, WPK5 exhibited the strongest inhibitory activity to FXIa (Figure 2B). WPK5 inhibited the amidolytic activity of FXIa to its substrate S-21(66) in a dose-dependent manner with an IC_50_ value of 978.20 ± 52.15 nM (Figure 2C,D). The activity of WPK5 on other serine proteases (trypsin, FXIIa, kallikrein, FXa, thrombin) were also measured. At 10-fold and 100-fold concentrations of the IC_50_ value against FXIa, WPK5 showed no or weak inhibitory activity to FXa, kallikrein, FXIIa and thrombin, but exhibited strong inhibitory activity to trypsin, with an IC_50_ of 10.55 μM (Table 1, Figure A1A,B). FXIa is a component of the intrinsic coagulation pathway, and the inhibition of FXIa would prolong the APTT and did not prolong the PT [30,46]. WPK5 dose-dependently prolonged APTT at the range of 0–160 μM. At a concentration of 80 μM, the APTT was 65.20 ± 0.66 s, which was 1.42-fold compared to the saline group (46.07 ± 0.28 s) (*p* < 0.0001). WPK5 did not prolong PT even at 160 μM (Figure 2E,F).

### 3.4. Improve WPK5 Potency by Loop Replacement

**Figure 3:**
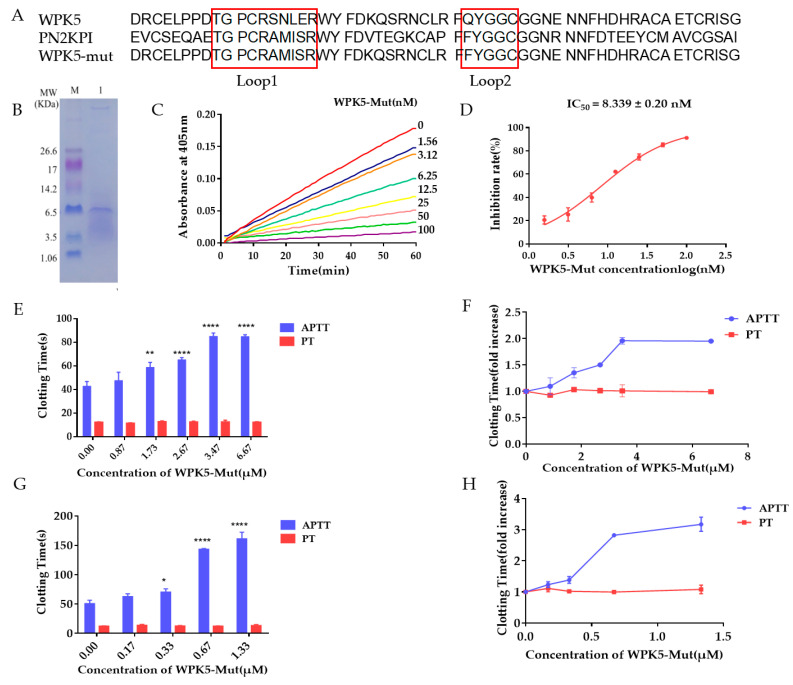
WPK5-Mut is a potent FXIa inhibitor. (**A**) Sequence information of WPK5, PN2KPI and WPK5-Mut. (**B**) Tricine-SDS-PAGE analysis of WPK5-Mut. Lane M: Marker, Lane 1: WPK5-Mut. (**C**) Inhibitory activity of WPK5-Mut to FXIa. Reaction started by addition of CS-21 (66) (250 μM) to a mixture containing WPK5-Mut (0–100 nM) and FXIa (0.5 nM). Substrate was hydrolyzed and monitored at 405 nm for 1 h. (**D**) Determination of IC_50_ of WPK5-Mut. Plot of inhibition rate (%) vs. log (WPK5-Mut concentrations) was fitted by nonlinear regression. (**E**,**F**) Effect of WPK5-Mut on APTT and PT of murine plasma. (**G**,**H**) Effect of WPK5-Mut on APTT and PT of human plasma. Data presented as mean ± SD of three independent experiments. **** *p* < 0.0001 versus vehicle, ** *p* < 0.01 versus vehicle, * *p* < 0.05 versus vehicle, analyzed by one-way ANOVA followed by Dunnett’s multiple comparison test.

WPK5 with an IC_50_ value of 978.20 ± 52.15 nM is not a potent FXIa inhibitor. PN2KPI, a famous Kunitz-type peptide secreted from platelet, showed good inhibitory activity against FXIa with an IC_50_ of 1.28 nM, and studies indicated that loop 1 and loop 2 in PN2KPI play a key role in its bioactivity [28,47,48]. We used the loop 1 (TGPCRAMISR) and loop 2 (FYGGC) in PN2KPI to replace the loop 1 (TGPCRSNLER) and loop 2 (QYGGC) in WPK5, and got a WPK5 mutant, named as WPK5-Mut. WPK5-Mut was expressed in *Pichia pastoris* GS115, and its in vitro anticoagulant activity was tested. As shown in Figure 3C,D, WPK5-Mut showed stronger inhibitory activity to FXIa with an IC_50_ of 8.34 ± 0.20 nM, which is about 100-fold higher potency than WPK5. Activity of WPK5-Mut to FXa, thrombin, kallikrein, trypsin or FXIIa was evaluated in enzyme assays. When the molar ratio of WPK5-Mut to FXIa is 50:1, the inhibition rate was 86.21%. While WPK5-Mut showed no or weak inhibitory activity to FXa, thrombin, kallikrein or FXIIa. When the molar ratio of WPK5-Mut to FXa or kallikrein or FXIIa is 775:1, the inhibition rate were 28.25%, 25.25%, 21.50%, respectively, indicating that WPK5-Mut have good selectivity on other proteases existing in the coagulation cascade. However, WPK5-Mut exhibited strong inhibition to trypsin, with an IC_50_ of 25.05 nM (Table 2, Figure A1C,D). The anticoagulant effect of WPK5-Mut was tested by APTT and PT in human plasma and murine plasma. In human plasma, WPK5-Mut prolonged APTT in a dose-dependent manner (Figure 3G,H), and doubled APTT at ~0.5 μM. It had no effect on PT even at a concentration of 1.33 μM (Figure 3E,F). In murine plasma, it also prolonged APTT in a dose-dependent manner; however, its potency was 7 times weaker (double APTT at ~3.5 μM) compare to that in human plasma.

### 3.5. WPK5-Mut Prevent FeCl_3_-Induced Carotid Artery Thrombosis in Mice

**Figure 4:**
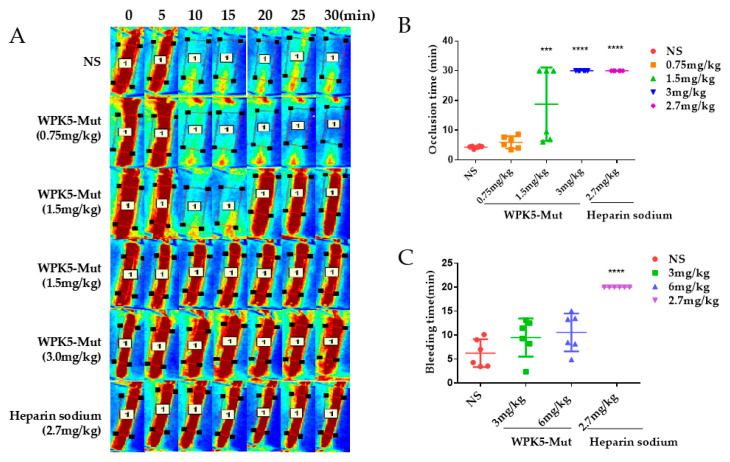
WPK5-Mut prevent FeCl_3_-induced carotid artery thrombosis in mice. Ten minutes before FeCl_3_-induce injury, WPK5-Mut (0.75, 1.5, and 3.0 mg/kg) or heparin sodium (2.7 mg/kg) was injected by tail vein, separately. (**A**) After treatment with 6% FeCl_3_, blood flow at region of interest (ROI) was monitored at 0, 5, 10, 15, 20, 25, and 30 min. (**B**) Effects of WPK5-Mut (0.75, 1.5, and 3.0 mg/kg) and heparin sodium (2.7 mg/kg) on occlusion time. (**C**) Effect of WPK5-Mut on bleeding time in mice. Fifteen min after the administration of WPK5-Mut (3, 6 mg/kg), heparin sodium (2.7 mg/kg) and the NS, a 3 mm-long tail tip was cut, and the tail was immersed immediately into saline at 37 °C. Accumulated bleeding time (including periods of re-bleeding) was recorded over 20 min. NS: normal saline. Data presented as mean ± SD (*n* = 6). *** *p* < 0.001 versus vehicle, **** *p* < 0.0001 versus vehicle, analyzed by one-way ANOVA, followed by Dunnett’s multiple comparison test.

To evaluate anti-thrombotic efficacy of WPK5-Mut in vivo, a FeCl_3_-induced carotid artery thrombosis model in C57BL/6J mice was used. Heparin sodium, which is a clinical anti-thrombosis medicine, was used as a positive control. As shown in Figure 4A,B, occlusion time was not significantly different between vehicle group (4.33 ± 0.16 min, mean ± SD, *n* = 6) and WPK5-Mut (0.75 mg/kg) treated group (5.92 ± 0.84 min, mean ± SD, *n* = 6). At a dose of 1.5 mg/kg, WPK5-Mut significantly prolonged occlusion time (18.75 ± 5.05 min, mean ± SD, *n* = 6). The occlusion did not happen within 30 min in three mice, while in other three mice, unstable thrombus formation was observed, as demonstrated that the blood vessel was gradually blocked at the beginning, and then flushed away by the blood flow and blocked again. At a dose of 3.0 mg/kg, occlusion did not happen within 30 min, which was similar to that of heparin sodium (2.7 mg/kg).

### 3.6. WPK5-Mut Did Not Show Bleeding Risk in Mice

To evaluate the bleeding risk of WPK5-Mut, a mice tail cutting assay was used. Doses were designed as 3 and 6 mg/kg, which represent 2 and 4 times the effective antithrombotic dosage, and heparin sodium (2.7 mg/kg) was used as a positive control. As shown in Figure 4C, WPK5-Mut at 6 mg/kg did not significantly prolong bleeding time (10.59 ± 3.96 min), compared to saline treated mice (7.35 ± 5.03 min), indicating that inhibition of FXIa did not impair the coagulation function. While heparin-sodium-treated mice cannot stop tail bleeding within 20 min (>20.00 ± 0.00 min, mean ± SD, *n* = 6).

## 4. Discussion

We disclosed five Kunitz-type peptides from the transcriptome of the salivary gland of the leech *Whitmania pigra*. Their inhibitory activity to FXIa were tested by the chromogenic substrate method. Among them, WPK5 showed strong inhibitory activity to FXIa. The potency of WPK5 was improved through loop-replacement strategy, and the mutant, WPK5-Mut, was further evaluated for the extensive bioactivity including in vitro anticoagulant activity and in vivo antithrombotic activity. WPK5-Mut prolonged APTT in both murine plasma and human plasma, and potently inhibited FeCl_3_-induced carotid thrombosis in mice. Interestingly, WPK5-Mut did not show significant bleeding risk even at 4 times the effective antithrombotic dosage in a mice tail cutting assay, indicating that WPK5-Mut is a promising candidate for the development of antithrombotic drug.

The salivary glands of leeches contain many anticoagulant and antiplatelet components, such as Theromin, a thrombin inhibitor from leech *Theromyzon tessulatum* [33]; Antistasin, a factor Xa inhibitor from the leech *Haementeria officinalis* [35]; Ornatins, a GPIIb-IIIa antagonist and platelet aggregation inhibitor from the leech *Placobdella ornata* [40]. FXIa inhibitory peptide from leeches has not been reported yet. Recently, we disclosed the transcriptome data of the salivary glands of leech *Whitmania pigra* [41]. In the present study, five Kunitz-type peptides were recognized from the transcriptome database. Among them, WPK5 exhibited the strongest inhibitory activity to FXIa with an IC_50_ of 978.20 ± 52.15 nM, and showed negligible inhibition to other proteases that exist in the coagulation cascade. FXIa is an intrinsic coagulation factor, and the inhibition of it prolong the APTT without affecting PT. The anticoagulant effect of WPK5 was evaluated by APTT and PT assay. At a dose of 80 μM, WPK5 prolonged 1.4-fold APTT, and did not prolong PT even at a dose of 160 μM. Based on our knowledge, this is the first FXIa inhibitory peptide from leeches. Some FXIa inhibitory peptides derived from other animals have been reported, such as rFasxiator [29] and Desmolaris [30]. rFasxiator is a Kunitz-type peptide from venom of the snake *Bungarus fasciatusm* with a IC_50_ of 1.5 μM, which is weaker than WPK5, while Desmolaris, a Kunitz-type protein from the salivary gland of a vampire bat, has poor selectivity (IC_50_ for FXIa = 1.32 nM, IC_50_ for FXa = 1.49 nM).

Domain swapping, loop replacement, and site-directed mutations are effective strategies to enhance the potency of proteins [49]. To improve the potency of WPK5, a loop replacement strategy was used. PN2KPI [47,48], a famous Kunitz-type peptide secreted from platelet, is a potent inhibitor of FXIa. The loop 1 and loop 2 of it can interact with the FXI catalytic domain, and prevent FXIa from interact with its substrate FIX, and affect the activation of FIX [28]. We used the loop 1 (TGPCRAMISR) and loop 2 (FYGGC) in PN2KPI to replace the loop 1 (TGPCRSNLER) and loop 2 (QYGGC) in WPK5, and got a WPK5 mutant named as WPK5-Mut. WPK5-Mut showed stronger inhibitory activity to FXIa with an IC_50_ of 8.34 ± 0.20 nM, which is about 100-fold higher potency than WPK5 in inhibitory activity to FXIa. The protease specificity data showed that WPK5-Mut effectively inhibited the activity of FXIa at a molar ratio of 50:1, with 86.21% inhibition. When the molar ratio of WPK5-Mut to kallikrein or FXIIa or FXa was 775:1, WPK5-Mut produced weak inhibitory activity with 25.25%, 21.50%, and 28.25%, respectively, and when the molar ratio of WPK5-Mut to thrombin was 1550:1, it showed no inhibitory activity, indicating that WPK5-Mut is selective for these enzymes. However, when the molar ratio of WPK5-Mut to trypsin was 39:1, WPK5-Mut could effectively inhibit the activity of trypsin, with an inhibition rate of 85.20%.

Although, the loop 1 and loop 2 of WPK5-Mut come from PN2KPI, and the inhibitory activity to FXIa of WPK5-Mut and PN2KPI is similar (8.34 ± 0.20 nM vs. 8.01 ± 3.92 nM), WPK5-Mut showed a stronger effect on murine plasma APTT than that of PN2KPI. WPK5-Mut prolonged APTT by 2 times at a concentration of 3.47 μM compared with saline treated group, while PN2KPI prolonged APTT by 1.5 times at the concentration of 19 μM [50]. The anticoagulant activity of WPK5-Mut varies according to different species. WPK5-Mut prolonged APTT in a dose-dependent manner both in murine plasma and human plasma. The efficacy of WPK5-Mut on human plasma (double APTT at ~0.5 μM) was stronger than that on murine plasma (double APTT at ~3.5 μM).

A FeCl_3_-induced carotid artery thrombosis model in mice was used to evaluate the antithrombotic effect of WPK5-Mut and PN2KPI [50] in vivo. WPK5-Mut at 3 mg/kg completely inhibited thrombosis, while PN2KPI did that at a higher dosage of 5.2 mg/kg. WPK5-Mut at 1.5 mg/kg partially inhibited thrombosis, while PN2KPI did that at a higher dosage of 2.6 mg/kg. Combination of both APTT assay and thrombosis model in vivo, we confirm that WPK5-Mut exerted stronger antithrombotic activity than PN2KPI. In previous studies, researchers thought that only the loops in Kunitz-type peptide affect its bioactivities. Here, from the difference of antithrombotic activity between WPK5-Mut and PN2KPI, we assume that not only the two loops, but also the scaffold of the peptide played a crucial role in the inhibition of thrombosis. It is worth to note that at 4 times the effective antithrombotic dosage, no significant bleeding was observed in a mice tail cutting assay, indicating that WPK5-Mut is a safer antithrombotic agent.

## 5. Conclusions

We identified a novel Kunitz-type FXIa inhibitor, WPK5, from leech *Whitmania pigra*. By loop replacement strategy, WPK5-Mut was obtained, and its ability to inhibit FXIa was increased by 100-fold compared to WPK5. WPK5-Mut prolonged plasma APTT in vitro, and significantly inhibited FeCl_3_-induced carotid artery thrombosis without bleeding risk. Taken together, WPK5-Mut is the first Kunitz-type FXIa inhibitor identified from leech *Whitmania pigra* with potent antithrombotic activity and could be a candidate for the development of clinical antithrombotic agent.

## Figures and Tables

**Table 1:** Protease Specificity of WPK5.

Enzyme	Inhibition (9.782 µM)	Inhibition (97.82 µM)
FXa	5.54%	4.88%
FXIIa	17.31%	42.16%
Kallikrein	57.26%	85.25%
Thrombin	0%	23.07%
Trypsin	44.40%	85.24%

Inhibition of WPK5 against FXa, FXIIa, kallikrein, thrombin, and trypsin was assessed in chromogenic substrate assay. WPK5 (9.782 µM, 97.82 µM) was incubated with the respective protease followed by addition of specific protease substrate. Results obtained from 3 independent experiments.

**Table 2:** Protease Specificity of WPK5-Mut.

Inhibtor	Molar Ratio	Inhibition, %
WPK5-Mut	50:1, inhibitor:FXIa	86.21
WPK5-Mut	775:1, inhibitor:FXa	28.25
WPK5-Mut	1550:1, inhibitor:Thrombin	−38.00
WPK5-Mut	775:1, inhibitor:Kallikrein	25.25
WPK5-Mut	39:1, inhibitor:Trypsin	85.20
WPK5-Mut	775:1, inhibitor:FXIIa	21.50

Inhibition of WPK5-Mut against FXIa, FXa, thrombin, kallikrein, trypsin and FXIIa assessed in a chromogenic substrate assay. Inhibitor was incubated with the respective protease at the indicated molar ratio followed by addition of a specific protease substrate. Results obtained from 3 independent experiments.

## Data Availability

The data that support the findings of this study are available from the corresponding author upon reasonable request.
